# Diagnosis of Deep Infiltrating Endometriosis Using Transvaginal Ultrasonography

**DOI:** 10.3389/fmed.2020.567929

**Published:** 2020-11-23

**Authors:** Shaoli Yin, Qi Lin, Fanhua Xu, Jinfeng Xu, Yujuan Zhang

**Affiliations:** ^1^Department of Ultrasound, Shenzhen People's Hospital, The Second Clinical Medical College of Jinan University, The First Affiliated Hospital of Southern University of Science and Technology, Shenzhen, China; ^2^Shenzhen Medical Ultrasound Engineering Center, Shenzhen, China

**Keywords:** transvaginal ultrasonography, deep infiltrating endometriosis (DIE), uterosacral ligament, intestinal, diagnosis

## Abstract

**Objective:** To evaluate the performance of transvaginal ultrasonography (TVS) in diagnosis of deep infiltrating endometriosis (DIE).

**Methods:** We retrospectively analyzed 198 patients with pathological diagnosis of DIE in our hospital from January 2017 to December 2019 and assessed the performances of pre-operative TVS diagnosis of DIE with regarding to sensitivity (SE), specificity (SP), positive predictive value (PPV), and negative predictive value (NPV), using the pathological diagnosis as the ground truth. We also characterized the ultrasonographic features of the DIE lesions.

**Results:** Among all the 198 cases, 170 cases were uterosacral ligament (USL) involvement, SE: 96.47% and SP: 85.71%; 79 cases were intestinal involvement, SE: 94.94% and SP: 94.96%; 57 cases were vaginal rectal septum (VRS) involvement, SE: 73.68% and SP: 94.33%; 20 cases were vaginal involvement, SE: 50% and SP: 97.21%; three cases were bladder involvement, SE: 66.7% and SP: 100%; nine cases were ureter involvement, SE: 55.56% and SP: 100%; and 10 cases were broad ligament involvement, SE: 10% and SP: 100%.

**Conclusion:** TVS showed high accuracy in diagnosing DIE.

## Introduction

Deep infiltrating endometriosis (DIE) is defined as subperitoneal invasion by endometriotic lesions that exceeds 5 mm in depth. DIE accounts for ~4–37% of all patients with endometriosis (1). DIE is strongly associated with high levels of pelvic pain, infertility, dysuria, dysmenorrhea, dyspareunia, and gastrointestinal distress, which may seriously affect the quality of life. However, because of insufficient understanding of the disease and lack of simple diagnostic tests, a timely diagnosis of DIE is often not available. It has been reported (2–5) that the average delay of DIE diagnosis (the time from the onset of the first symptom to the clinical diagnosis of endometriosis) was 10 years in Germany, 7 years in Brazil, 11.7 years in the US, and 8 years in the UK. It has been challenging to detect DIE by ultrasound for many years (6, 7). The purpose of this study was to explore the performance of transvaginal ultrasonography (TVS) in DIE diagnosis.

## Materials and Methods

### Ethics Statement

The Medical Science Ethics Committee of Shenzhen People's Hospital approved this study (No. 2018100). Each patient or an appropriate family member provided informed written consent for the collection of clinical materials.

### Patients

This was a retrospective study. From January 2017 to December 2019, a total of 198 patients suspected of DIE were included in the study, and all of them underwent laparoscopic surgery and histological examinations. All patients were enrolled from Shenzhen People's Hospital. The indications for TVS were chronic pelvic pain, infertility, dysmenorrhea, dyspareunia, and dyschezia.

### Inclusion and Exclusion Criteria

Inclusion criteria: (1) patients with clinically suspected DIE based on medical history and findings; and (2) patients who agreed to accept surgical treatment for DIE.

Exclusion criteria: (1) women who left this study cohort for personal reasons; (2) women who got pregnant during waiting for surgery; (3) patients with severe intestinal gas interference, which could affect the results; and (4) patients who cancelled their surgery.

### Procedure

All TVS scans were performed by one examiner who had received professional training. The examiner was blinded to the physical examination and previous imaging examination results but was aware that the women were being evaluated for chronic pelvic pain and that endometriosis was suspected. All TVS examinations were performed within 2 weeks of surgery. All women were examined in the lithotomy position with either a GE E8 (GE Healthcare Ultrasound, USA) or Philips IU22 (Philips IU22, USA) scanner equipped with a 5–9-MHz or C10-3 transducer for transvaginal visualization.

### TVS Technology

TVS examinations were performed with ultrasound transmission gel in the probe cover to create a stand-off to visualize the near-field area. First, the examiner observed whether the uterine muscle layer and both ovaries were associated with adenomyosis or chocolate cysts, which are frequently associated with DIE. Then, the probe was positioned on the perineum and slowly inserted into the vagina for observation of the vaginal wall and vaginal fornix, vaginal rectal septum (VRS), uterosacral ligament (USL), broad ligament, and intestinal canal. Moreover, according to the consensus of the International Organization for the Analysis of Deep Endometriosis (6), a scan of the urinary system (bladder, kidney, and ureter) was routinely performed. During the examination, the examiner paid great attention to sites with tenderness and observed whether the sliding sign of the uterus was impaired. It is an important step to evaluate the mobility between the posterior bladder dome and anterior uterine serosa via a bimanual examination combining gentle pushing of the probe and abdominal pressure with the free hand. The sliding sign was considered positive when free movement was observed between the bladder and uterus. Bimanual examination was also performed to assess ovarian mobility. By applying pressure, the ovaries could slide freely against the pelvic sidewalls. The sliding sign could also be used to assess the free mobility between the posterior uterus wall and the anterior rectal wall.

All of the involved targets, especially painful sites, were evaluated in multiple scanning planes by rotating the transducer. The lesion size was measured in three orthogonal planes.

### Golden Criteria of DIE

In accordance with Bazot et al.'s criteria (8), deep pelvic endometriosis was diagnosed in one of the following isolated or combined circumstances:

1. Endometrial tissue (endometrial glands and stroma) was found in at least one resected lesion (9);
2. Direct visualization of a deep pelvic endometriotic lesion showed only fibrosis at biopsy; if the lesion was not biopsied, at least one other site of endometriotic involvement was proven histologically;
3. There was complete cul-de-sac obliteration secondary to unresectable endometriosis (because of an absence of perioperative consent for extensive surgery). We considered that in such cases, deep retrocervical endometriosis was present beneath the peritoneum (10).

### Statistical Analysis

All statistical analyses were performed with the Statistical Package for Social Science version 25.0 (SPSS Inc, Chicago, IL). The results were expressed as the mean ± standard deviation or proportions. The definitive diagnosis of DIE was defined based on the surgical findings and histopathologic results. With the surgical and pathological results as the gold standard, we calculated the sensitivity, specificity, positive predictive value (PPV), and negative predictive value (NPV) of TVS in the diagnosis of DIE in different sites. The positive and negative likelihood ratios (LR+ and LR–), as well as the accuracy, were also calculated. A consistency check was used to calculate the overall levels of agreement between TVS and surgical findings for DIE. The strength of concordance reflected by the kappa coefficient is as follows: 0.00, “poor”; 0.00–0.20, “slight”; 0.21–0.40, “fair”; 0.41–0.60, “moderate”; 0.61–0.80, “substantial”; and 0.81–1.00, “almost perfect.” A two-sided *P*-value of <0.05 was considered statistically significant. In patients with bilateral USL involvement identified at surgery, we classified the findings as false negatives if only one side of the USL was diagnosed correctly.

## Results

### Patient Data

After applying the inclusion and exclusion criteria, a total of 198 women suffering from chronic pelvic pain were included in the study. The enrolled women were 21–51 years of age (mean, 35.36 years; SD, 7.6 years).

### Surgical and TVS Findings

A total of 198 cases of pelvic DIE were confirmed by surgery and pathology, including 170 cases of USL involvement, of which 164 cases were correctly diagnosed by TVS; 79 cases of intestinal involvement, of which 75 cases were correctly diagnosed by TVS; 57 cases of VRS involvement, of which 42 cases were correctly diagnosed by TVS; 20 cases of vaginal involvement, of which 10 cases were correctly diagnosed by TVS; 3 cases of bladder involvement, of which 2 cases were correctly diagnosed by TVS; 9 cases of ureter involvement, of which 5 cases were accurately diagnosed; and 10 cases of broad ligament involvement, of which 1 case was diagnosed correctly before surgery (Table 1).

**Table 1:** Performance of TVS for the pre-operative diagnosis of endometriosis in 198 patients with surgical results as the gold standard.

**Location**	**Sensitivity**	**Specificity**	**PPV**	**NPV**	**LR+**	**LR–**	**Accuracy**
Uterosacral ligament (*n* = 170)	96.47% (164/170)	85.71% (24/28)	97.62% (164/168)	80.00% (24/30)	6.75	0.04	94.95%
Intestinal (*n* = 79)	94.94% (75/79)	94.96% (113/119)	92.59% (75/81)	96.58% (113/117)	18.84	0.05	94.95%
Vaginal rectal septum (*n* = 57)	73.68% (42/57)	94.33% (133/141)	84% (42/50)	89.86% (133/142)	12.97	0.28	88.38%
Vagina (*n* = 20)	50% (10/20)	97.21% (174/179)	66.67% (10/15)	94.57% (174/184)	17.92	0.51	92.93%
Bladder (*n* = 3)	66.67% (2/3)	100% (195/195)	100% (2/2)	99.49% (195/196)	#	0.33	99.49%
Ureter (*n* = 9)	55.56% (5/9)	100% (189/189)	100% (5/5)	97.93% (189/193)	#	0.44	99.49%
Broad ligament (*n* = 10)	10% (1/10)	100% (188/188)	100% (1/1)	95.43% (188/197)	#	0.90	95.45%

*PPV, positive predictive value; NPV, negative predictive value; LR+, positive likelihood ratio; LR–, negative likelihood ratio; #, not available*.

Table 2 shows the levels of agreement between TVS and surgical findings for deep endometriosis in different sites. The agreement was almost perfect for intestinal involvement. There was substantial agreement between TVS and surgery in identifying USL involvement, VRS endometriosis, bladder involvement, and endometriosis of the ureter. The agreement was moderate for endometriosis of the vagina. However, the agreement was slight for broad ligament endometriosis.

**Table 2:** Levels of agreement between TVS and surgical findings for deep endometriosis in different sites.

**Location**	**Kappa**	***p***
Uterosacral ligament	0.8	0.000
Intestinal	0.895	0.000
Vaginal rectal septum	0.706	0.000
Vagina	0.531	0.000
Bladder	0.798	0.000
Ureter	0.705	0.000
Broad ligament	0.174	0.000

**Figure 1:**
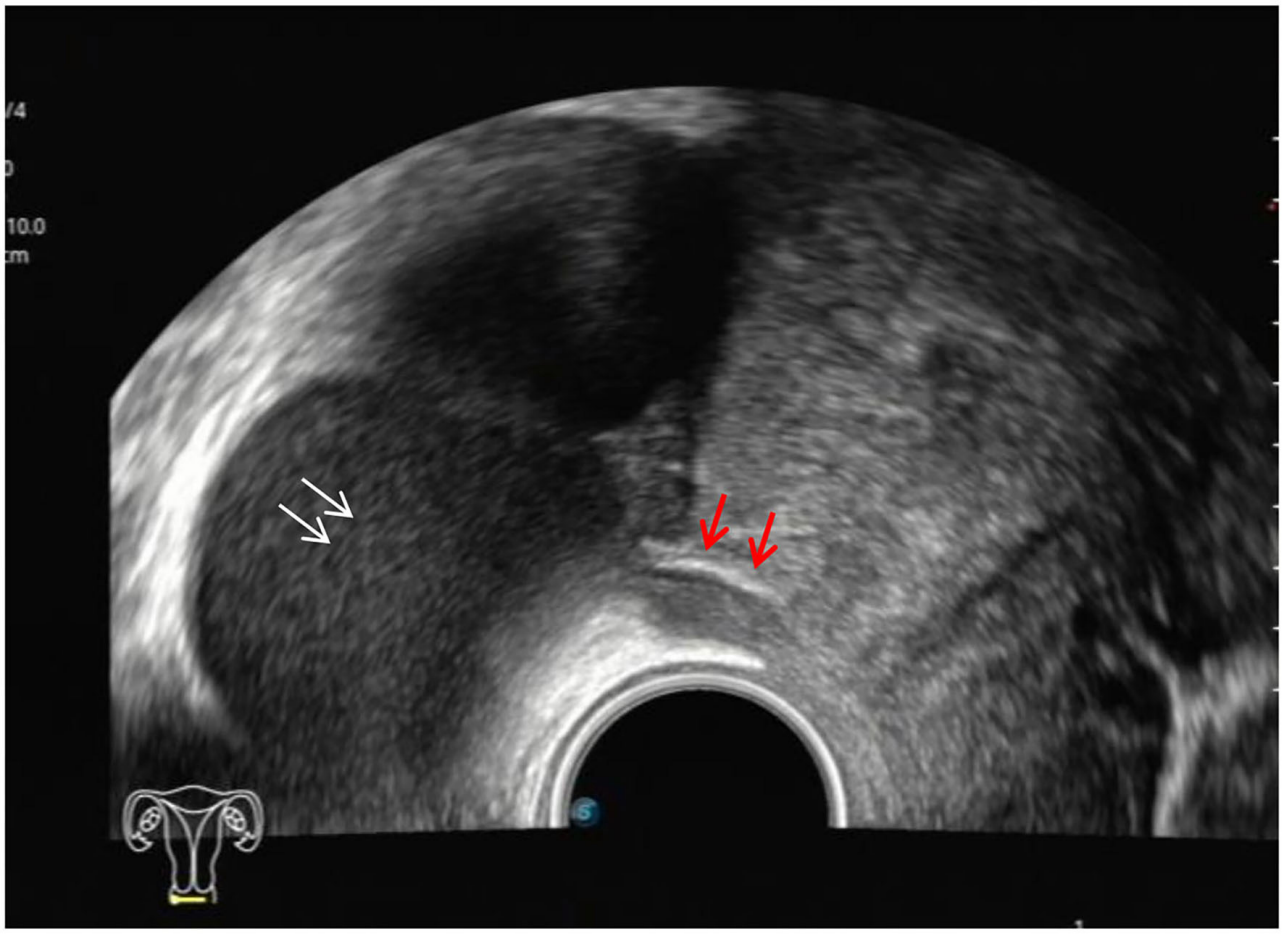
Red arrows, hypoechoic thickening of the right USL; white arrows, right ovary with chocolate cyst.

**Figure 2:**
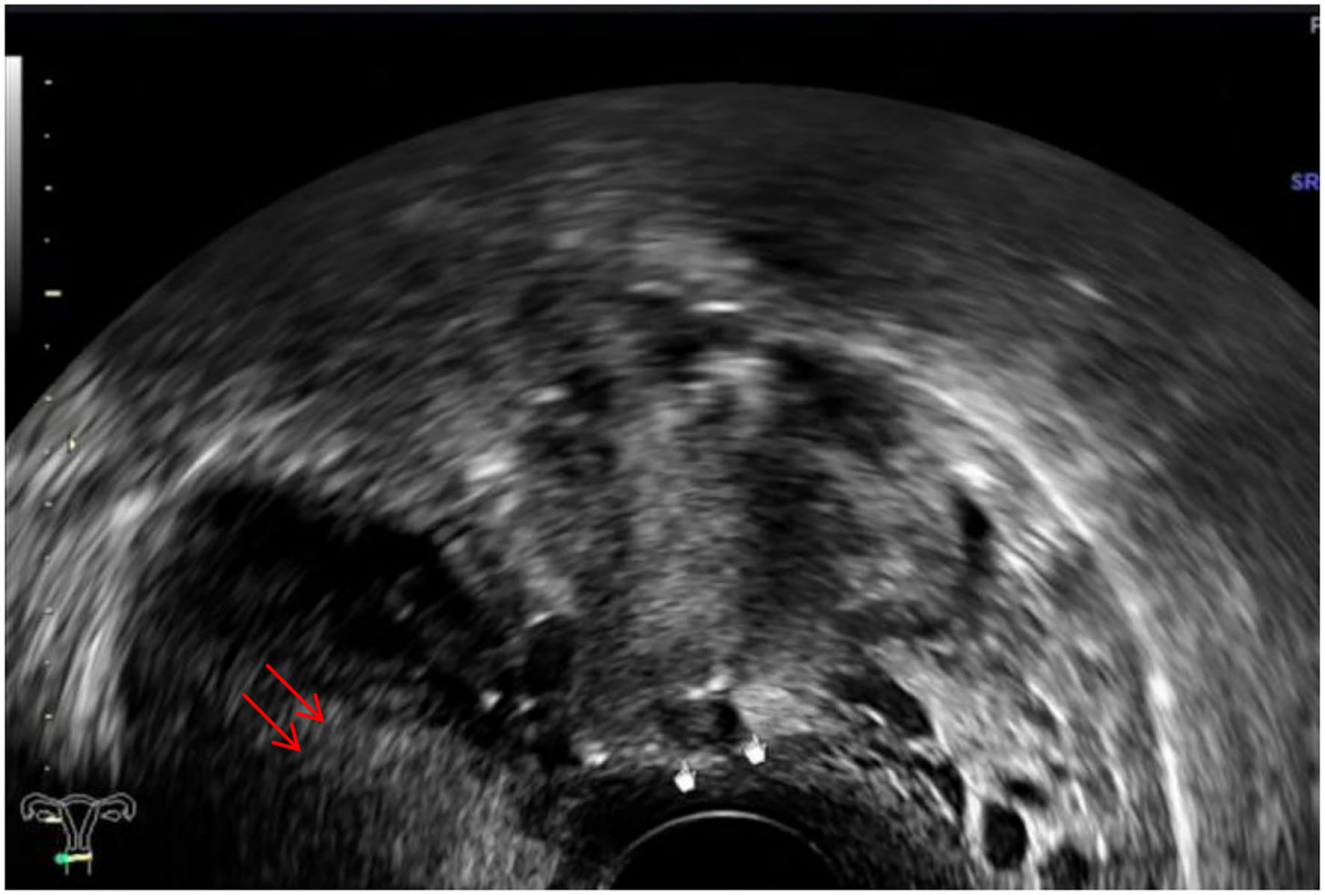
White mark, hypoechoic nodular lesions of the right USL; red arrows, uterus.

**Figure 3:**
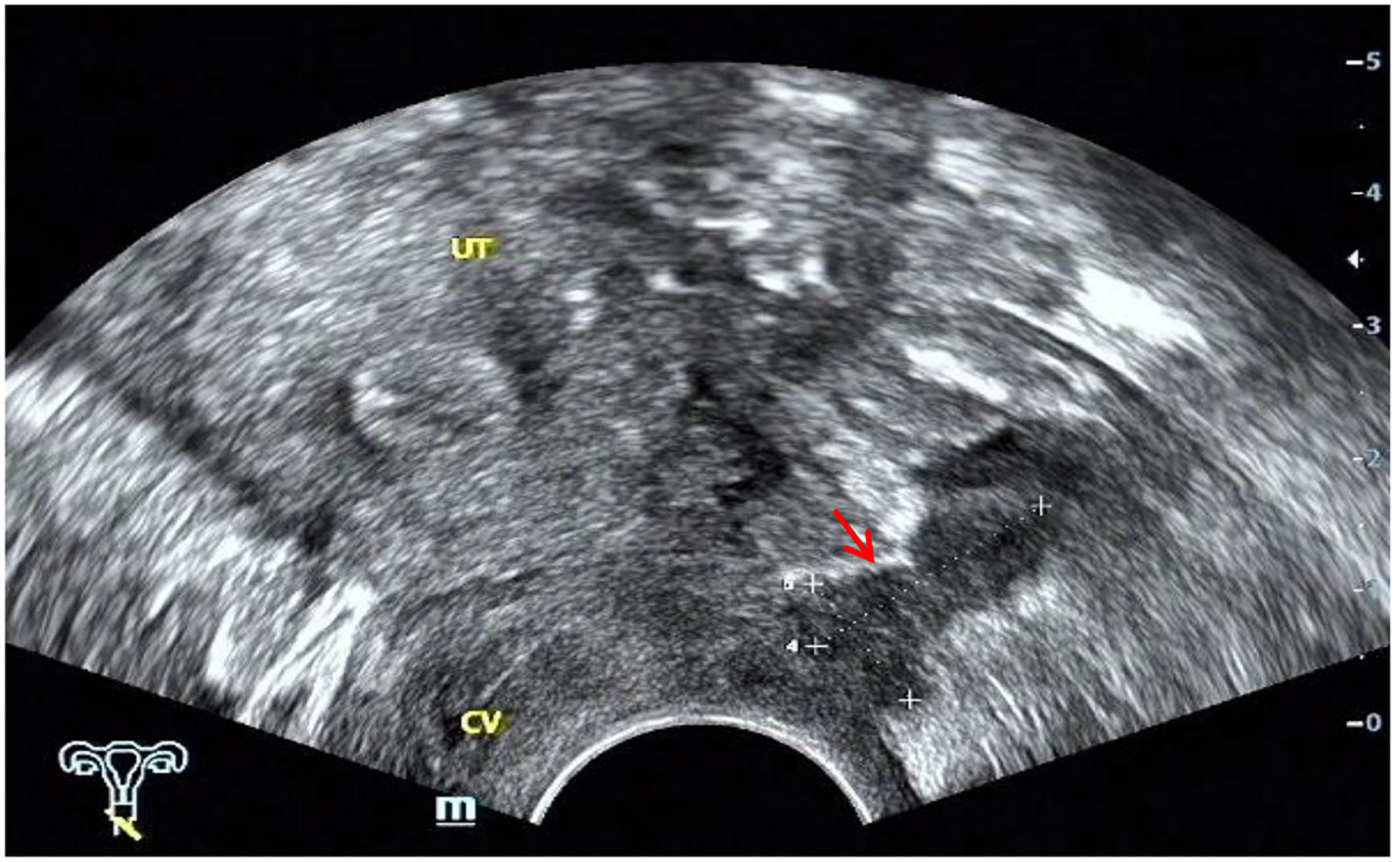
Red arrow, cable-shaped hypoechoic lesion along the left USL; CV, cervix; UT, uterus.

**Figure 4:**
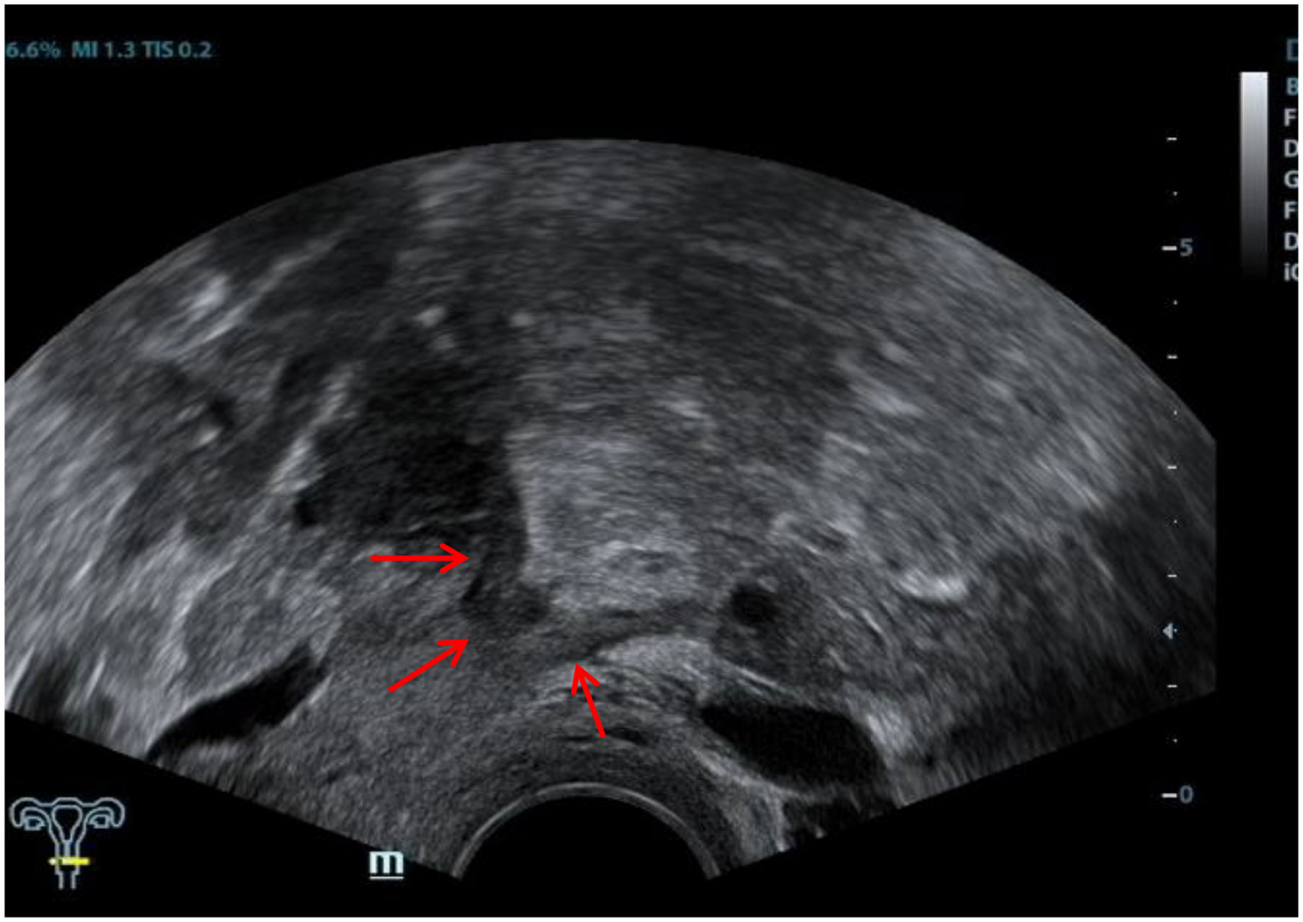
Red arrows, thickening of both the USL and the torus uterinus.

**Figure 5:**
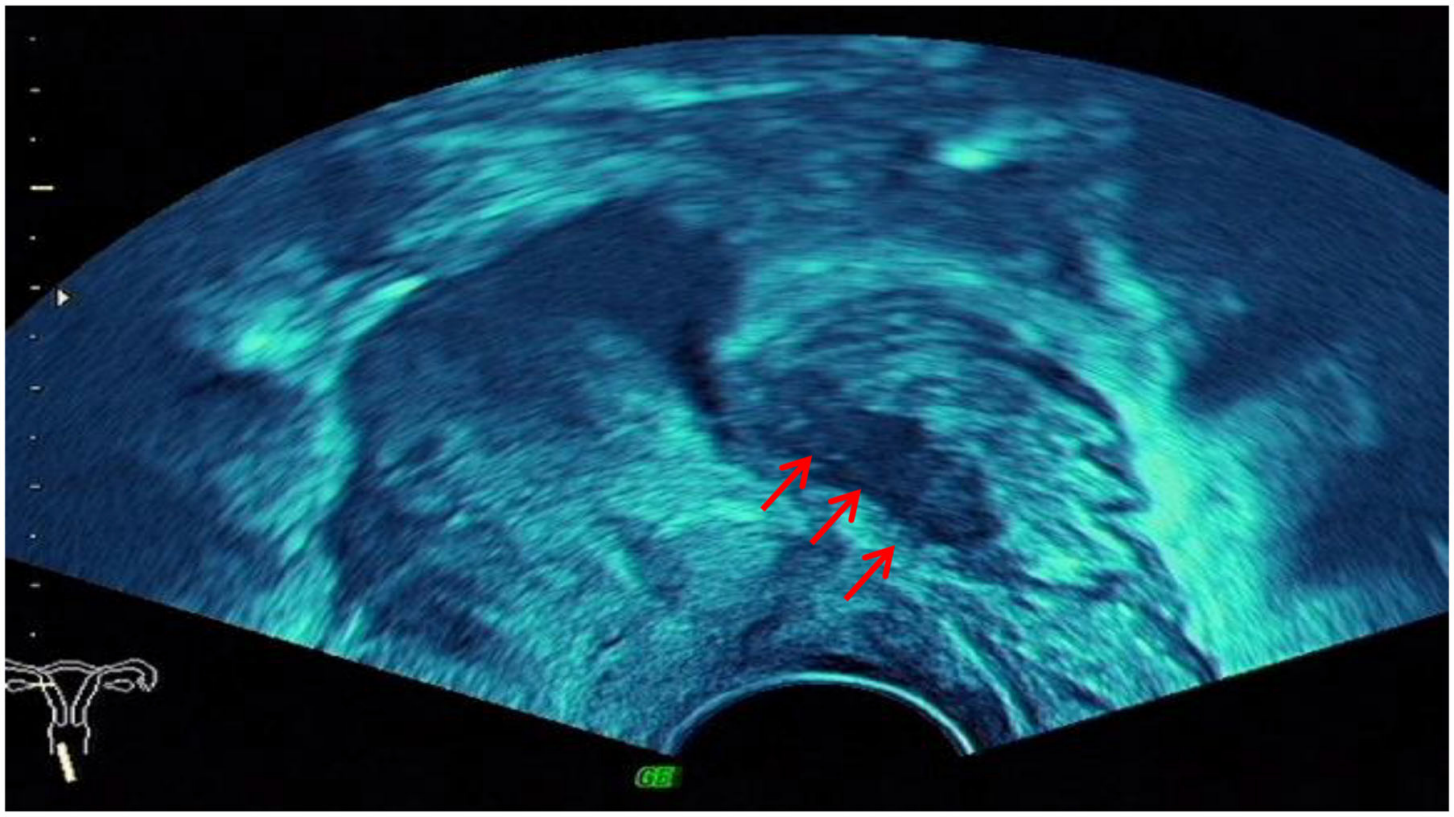
Red arrow, “Indian headdress” signs of intestinal lesions.

**Figure 6:**
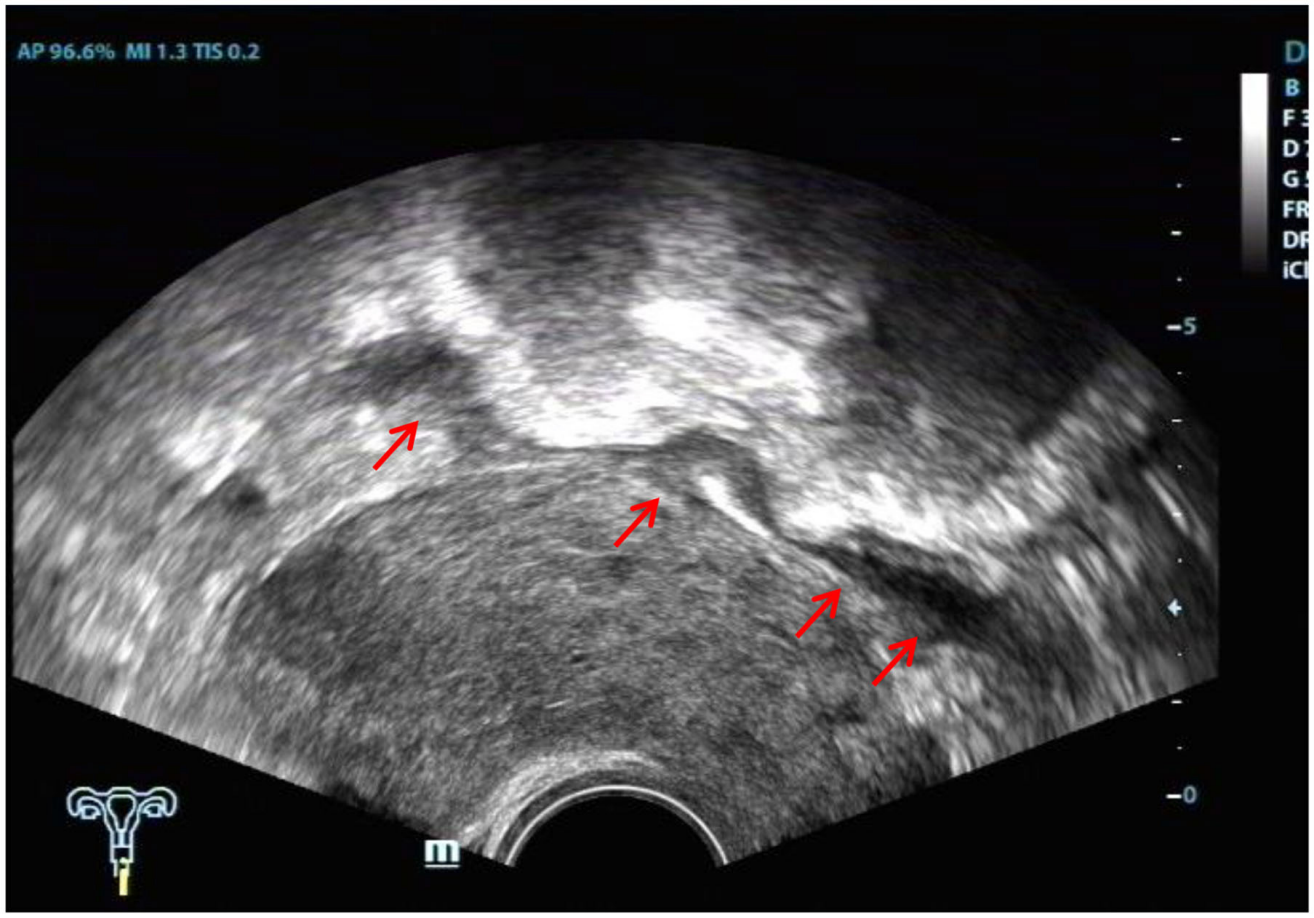
Red arrows, segmental thickening of multiple intestinal lesions.

**Figure 7:**
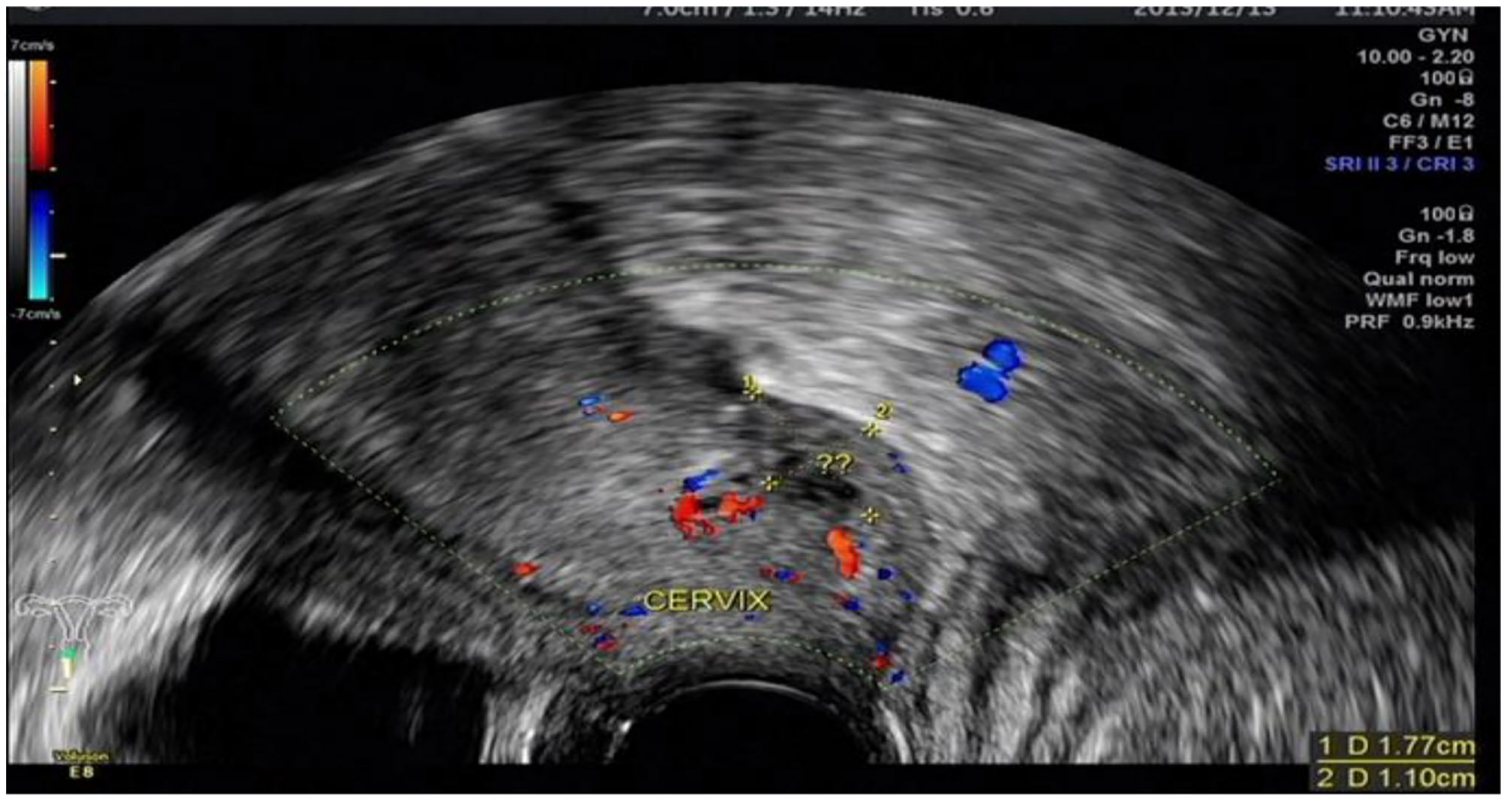
DIE lesion of the vaginal fornix, with slightly rich blood flow.

**Figure 8:**
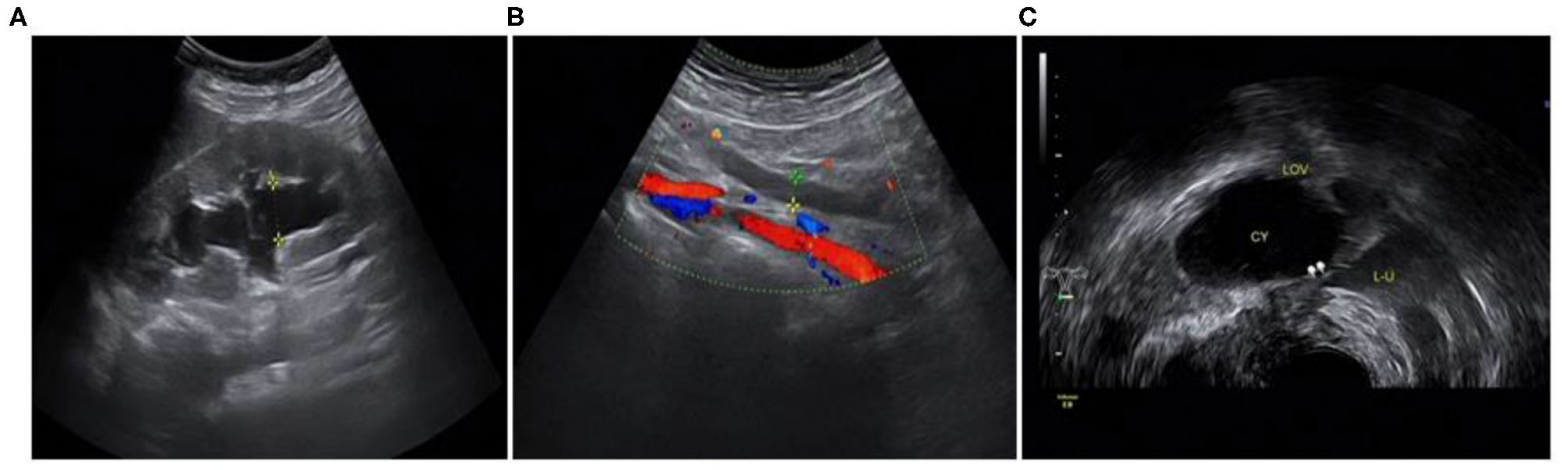
Involvement of the left ureter: **(A)** hydronephrosis of the left kidney; **(B)** dilated proximal ureter; **(C)** narrow involved lower part of the left ureter.

**Figure 9:**
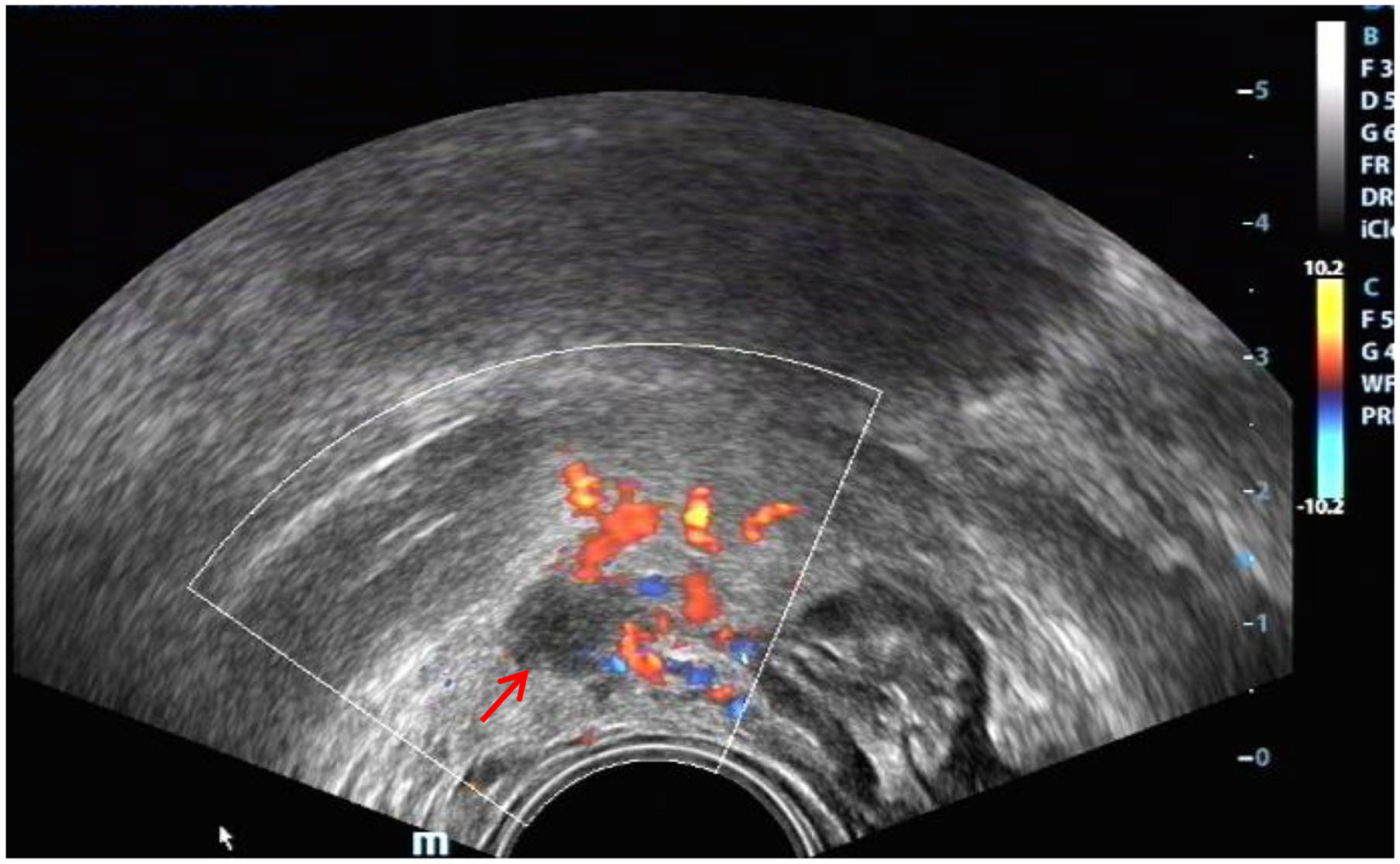
Red arrow, DIE lesion of the right broad ligament, a hypoechoic zone with an irregular shape.

The ultrasound characteristics of the DIE lesions in each affected location are summarized as follows (Table 3). There were multiple manifestations when the USL was affected; 50.61% (83/164) showed local thickening of the ligament root, reduced echo, and stiffening (Figure 1); 27.44% (45/164) demonstrated a nodular lesion with a circular or star shape (Figure 2); 16.46% (27/164) presented cable-shaped, low-echo lesions along the USL, with regular or irregular edges (Figure 3); and 5.49% (9/164) showed that bilateral USL and the torus uterinus were thickened (Figure 4). Sonograms of the affected intestines were characteristic, with 32% (24/75) showing “Indian headdress” signs (Figure 5), and 68% (51/75) showing segmental thickening (Figure 6) with low echoes, which were characterized by infiltration starting from the serosa layer, and multiple lesions could be found. All (42/42) of the involved lesions of the VRS presented nodular or long hypoechoic zones between the posterior vagina and rectum, with visible microcystic structures. The vaginal wall lesions were mostly nodular and hypoechoic with or without small non-echoic areas (7/10). Blood flow in the lesions at the vaginal fornix was more abundant than in other sites (6/10) (Figure 7). The lesions of the bladder wall were nodular or lumpy; the two cases were both located in the posterior wall of the bladder, and the peripheral echoes tended to be slightly enhanced, probably with small echoless areas inside. Five cases of ureteral involvement were accompanied by hydronephrosis of the kidney and the proximal ureter. Follow-up scans of the dilated ureter revealed narrowing of the distal ureter (Figure 8). Broad ligament involvement presented an irregularly shaped hypoechoic zone on the right or left side of the uterus without a capsule and might be accompanied by echo-enhanced and thickened peripheral peritoneum (Figure 9).

**Table 3:** Ultrasonographic features of DIE lesions in different sites.

**Site**	**Ultrasonographic feature**
Uterosacral ligament	Local thickening of the ligament root, reduced echo, and stiffening (83/164); a nodular lesion with a circular or star shape (45/164); cable-shaped, low-echo lesions along the USL, with regular or irregular edges (27/164); thickening of bilateral USL and the torus uterinus (9/164).
Intestinal	“Indian headdress” signs (24/75); segmental thickening with low echoes characterized by infiltration starting from the serosa layer (51/75).
Vaginal rectal septum	Nodular or long hypoechoic zone between the posterior vagina and rectum with visible microcystic structure (42/42).
Vagina	Nodular hypoechoic lesions with or without small non-echoic areas (7/10); abundant blood flow (6/10).
Bladder	Nodular or lumpy lesions located in the posterior wall of the bladder with peripheral echoes that might be slightly enhanced, probably with small echoless areas inside (2/3).
Ureter	Lesions accompanied by hydronephrosis of the kidney and the proximal ureter. Narrowing of the distal ureter (5/9).
Broad ligament	Irregularly shaped hypoechoic zone on either side of the uterus without capsule likely accompanied by enhanced and thickened peripheral peritoneal echoes (1/10).

## Discussion

As the first-line diagnostic method in gynecology, ultrasound examination has always been unsatisfactory in diagnosing DIE, and literature on the application of ultrasound in the diagnosis of DIE are rare in China. The reasons for the current poor ultrasound diagnosis rate are as follows: (1). ultrasound doctors do not know enough about DIE and lack diagnostic experience; (2) involved DIE lesions are usually located in the posterior cervical region with a narrow range, complex structures, and blurred boundaries; (3) during the ultrasound examination, affected areas are easily ignored or difficult to clearly show, such as the USL, the VRS, and the vagina; and (4) these easily affected areas are mostly near the intestine, and intestinal gas interference affects ultrasound imaging. To address these disadvantageous factors, we devised several strategies prior to the study: placing some coupling agent in the condom to create an acoustic window; obtaining a full understanding of the anatomy of the affected areas; and developing inspection specifications. The results of these preparative steps showed that ultrasound had great value in diagnosing the affected areas of DIE, especially the USL, intestine, and ureter. It also indicated that the mentioned methods could improve the ultrasound diagnosis rate of DIE.

We have used a few strategies during the study in order to reduce the likelihood that there is discrepancy of DIE location diagnosed with ultrasound scan and the actual findings during laparoscopy (and subsequent biopsy). First, the ultrasound doctor has abundant diagnostic experience and good understanding of the anatomy of the commonly affected sites. Second, during the examination, we describe the specific location of the DIE lesion, adjacent relationship between the lesion and surrounding tissues, and whether the lesion adheres to other parts. Third, during the surgery, attention was paid to the structure adjacent to the lesions and then checked. Finally, after we have surgically removed the lesion, we gave a detailed label which included information of its exact location, origin etc.

### Uterosacral Ligament DIE

**Figure 10:**
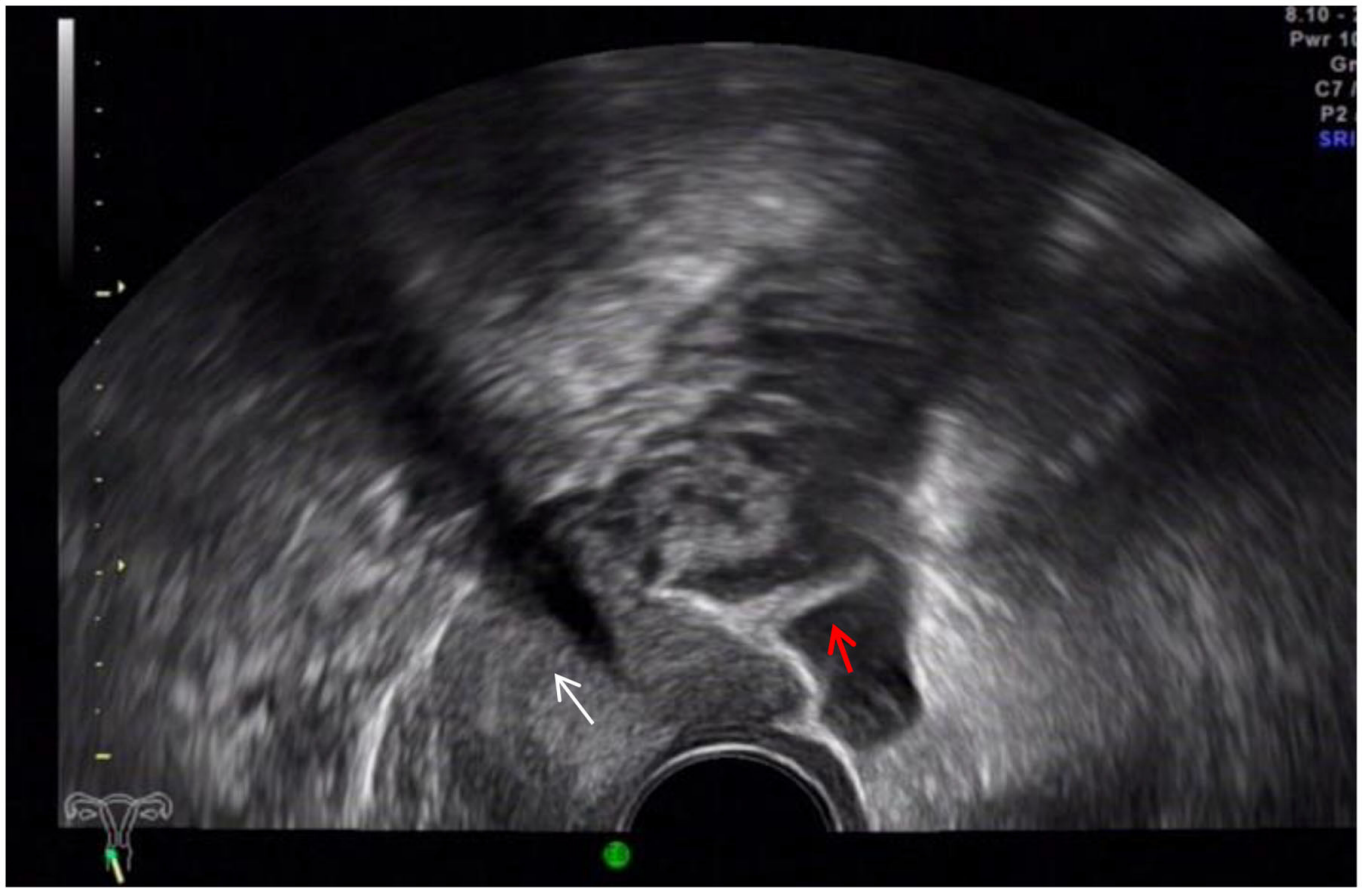
Red arrow, normal left USL; white arrow, cervix.

Many scholars (8, 11) consider visualization of the USL by ultrasound to be impossible. As early as 1996, it was reported (12) that transrectal ultrasonography could observe normal or affected uterine ligaments. In our experience, TVS can show USL in some healthy women, especially when the pelvic cavity is accompanied by a small amount of fluid. The normal USL appears as an arcuated and hyperechoic structure around the rectum from the upper side of the back of the cervix to the sides (Figure 10). Lesional involvement in the USL presented as hypoechoic nodules, cable-like echoes, local thickening of the root or thickening of bilateral USL and the arch. In this study, the sensitivity of ultrasound diagnosis was 96.47%, higher than the results of other reports (8, 13). At the same time, the specificity of ultrasound-diagnosed USL lesions in this study was 85.71%, lower than other statistical indicators. The main reason for this was the fact that three cases diagnosed as unilateral involvement were classified into the false negative group in the study, which has not been encountered in other studies.

### Intestinal DIE

**Figure 11:**
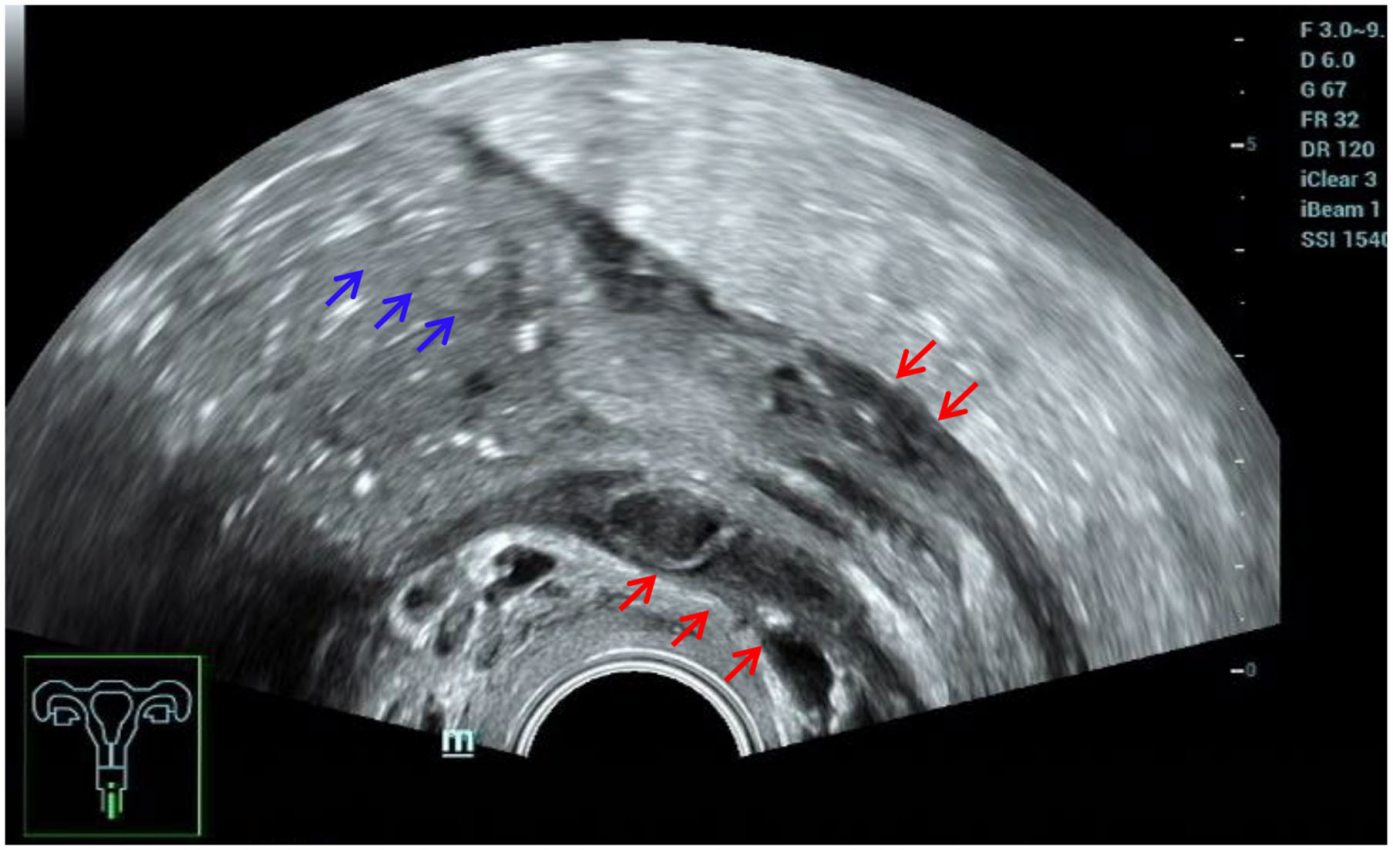
Misdiagnosed ulcerative colitis. Red arrows, thickening of both the anterior and posterior walls of the affected colon; blue arrows, proximal bowel obstruction.

Consistent with the results of numerous studies (3, 14), ultrasound played an important role in the diagnosis of intestinal DIE. In this study, the sensitivity was 94.94%, and the specificity was 94.96%. This is mainly because the ultrasound images of intestinal DIE lesions were very characteristic and easy to identify, and all patients in this study made bowel preparations, which was conducive to improving the detection rate of intestinal lesions. Five false-positive cases were actually adhesions between the posterior cervical lesion and the rectum, without invading the intestinal wall. Another false-positive case turned out to be ulcerative colitis after surgery. Retrospective analysis of the ultrasound images revealed that both the anterior and posterior walls of the affected colon were thickened rather than the segmental thickening of the anterior wall of intestine DIE, accompanied by proximal bowel obstruction (Figure 11). Although the diagnostic performance was good, we should also pay much attention to the differential diagnosis of other intestinal diseases, as some scholars have suggested (15).

**Figure 12:**
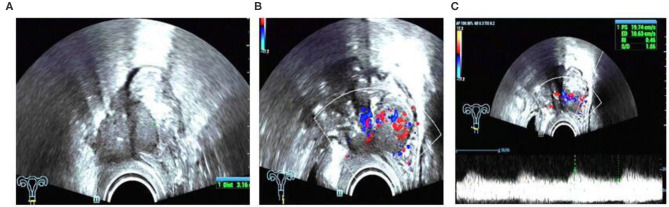
Intestinal endometriosis with malignant transformation: **(A)** hypoechoic mass with blurred borders and an irregular shape; **(B)** abnormally abundant blood flow; **(C)** high-speed low-resistance blood flow spectrum of the mass.

In this study, we found a case of intestinal endometriosis with malignant transformation pre-operatively. The patient had undergone hysterectomy due to DIE 1 year prior and presented with bloody stool 3 months after surgery. TVS showed a low-echo mass with a diameter of approximately 40 mm on the right side of the vaginal stump and the anterior wall of the rectum. The boundary was not clear, and the shape was irregular. In addition, abundant blood flow and a high-speed, low-resistance blood flow spectrum can be seen in the mass (Figure 12). Ultrasound examination can show the characteristics of malignant lesions in the intestinal tract, but distinguishing it from primary or metastatic cancer of the intestine is difficult. For patients with a history of endometriosis, especially those using hormone replacement therapy, the possibility of endometrial malignant transformation should be considered. Endometriotic lesions of the intestine mostly grow inward from the serosa layer, which is helpful for differential diagnosis. Ultrasound has a high diagnostic rate for intestinal DIE lesions; however, it cannot be compared with rectal ultrasound endoscopy when evaluating the depth of intestinal involvement and determining the distance from the anus (8).

### VRS and Vaginal DIE

Our study showed that the specificity of VRS and vaginal DIE was good while the sensitivity was low at 73.68 and 50%, respectively, when compared with that of other involved sites. The main reason for these results was that the ultrasound features of the lesions were not very characteristic, and the ultrasound diagnosis of small or single DIE lesions was limited. Ultrasound easily missed nodules of the vaginal wall because of vaginal wall collapse and interference with vaginal gas. The method of placing some couplant in the probe condom could improve imaging quality and be conductive to demonstrating vaginal wall lesions.

**Figure 13:**
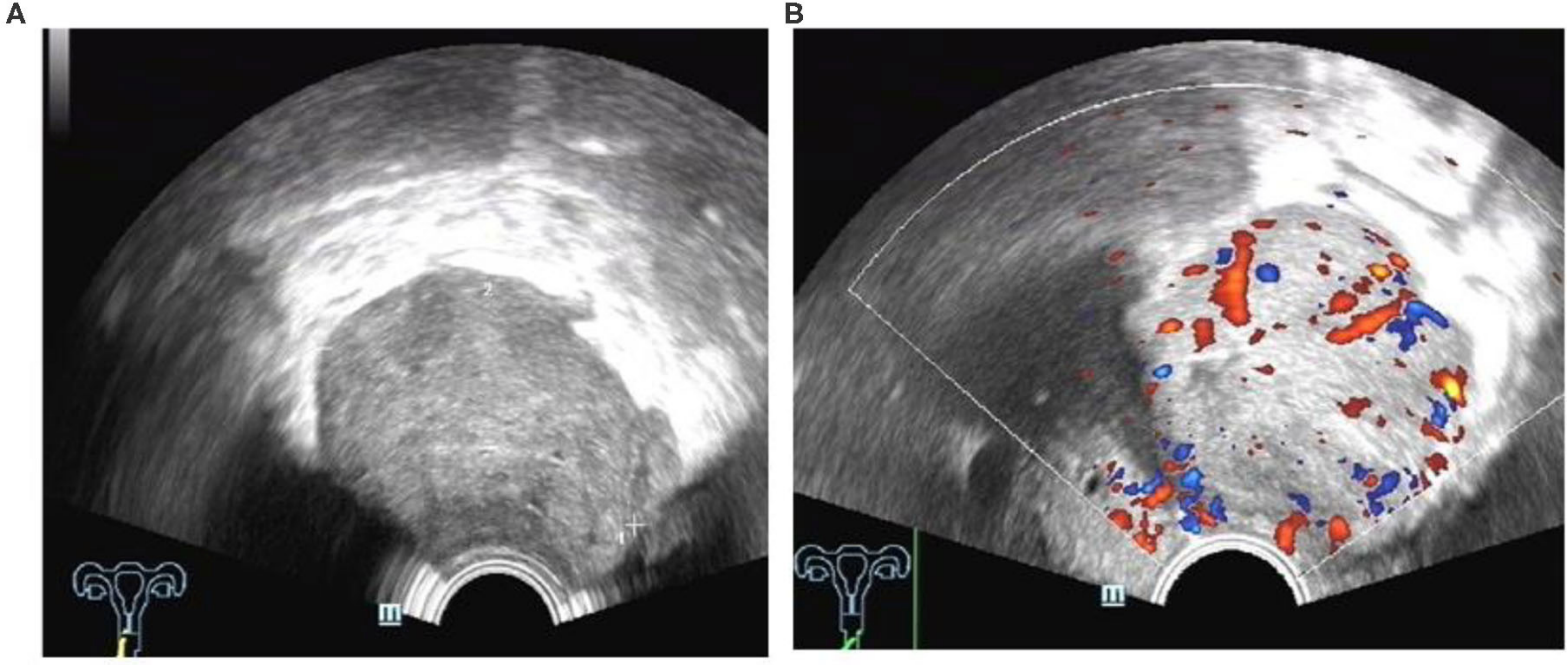
Lesion of the vaginal rectal septum with malignant transformation: **(A)** an irregular mass with blurred borders located at the vaginal rectal septum; **(B)** fairly abundant blood flow in the mass.

A case of VRS endometriosis with malignant change was detected before surgery. TVS showed a mass with a diameter of approximately 60 mm, a blurred border and an irregular shape. The mass presented abundant blood flow (Figure 13), which can be a crucial clue for differential diagnosis.

### Bladder DIE

In the study, there were just three cases of bladder involvement. Consistent with the results of other studies (8, 16), the results of this study indicate that ultrasound is not very sensitive in diagnosing bladder endometriosis. The main reason is that it is difficult to characterize lesions of the bladder unless the patient has typical clinical manifestations of periodic urinary frequency and dysuria or evidence of endometriosis at other sites, such as uterine scars. The two cases that were correctly diagnosed in this study were detected due to a comprehensive analysis of the patient's medical history. One case located at the top of the bladder was missed due to overfilling of the bladder and the small size of the lesion. The ultrasound image characteristics of the bladder DIE in the study were as follows: single lesions were located in the posterior wall of the bladder, while when scars on the anterior wall of the uterus invaded the bladder, it was at the anterior wall. Abnormal hypoechoic nodules, round or clumped and with clear borders, could be seen. Sometimes small anechoic areas can be seen in typical lesions. Color Doppler imaging did not show significant blood flow. These characteristics can be used to distinguish between bladder tumors and cystitis. For cases without specific clinical manifestations, it is difficult for ultrasound doctors to make a differential diagnosis based on the sonogram alone, requiring them to prompt further examination to confirm the diagnosis.

### Ureteral DIE

The early diagnosis of ureteral DIE is of great importance, because timely intervention can prevent the loss of renal function caused by ureteral obstruction. Usually, ureteral endometriosis is associated with other DIE lesions. The incidence of ureteral endometriosis is estimated to be 0.08–12% of all women with DIE. However, for endometriosis involving the USL, there is greater ureteral involvement, and the prevalence can reach 35% (17). Therefore, ureteral involvement should be kept in mind when USLs are also clinically involved (17).

The consensus of the International Organization for the Analysis of Deep Endometriosis (6) states that in view of the lack of specific clinical manifestations of urinary DIE and to prevent the loss of renal function, a complete and comprehensive scan of the urinary system should be performed in all patients with endometriosis. Histologically (18), according to the presence of the endometrial glands and interstitium in the muscular layer or even the submucosa of the ureteral wall, the endometriosis is divided into two types, extrinsic and intrinsic, with an external to internal incidence ratio of approximately 4:1. In our study, pathological results showed that there were eight extrinsic and two intrinsic cases, which coincided with the literature.

Five cases received the correct pre-operative diagnosis. After finding hydronephrosis, we followed up and scanned the dilated ureter up to the stenosis, where it was mostly compressed, with a tortuous path, and hypoechoic lesions can be seen around it. In the study, the five cases of ureteral involvement were single lesions located in the middle and lower ureter after crossing the iliac vessels. The four missed cases' surgical findings showed that the lesions were just invading the surface of the ureter, which made them hard to detect by TVS.

In short, a comprehensive urinary scan of patients with DIE can be helpful to detect ureteral lesions, as mentioned in other published literature (19). However, for cases without hydronephrosis of the kidney or lesions that are just invading the surface of the ureter, such as the missed cases in this study, more advanced skills are needed, and further studies are also required to better demonstrate the diagnostic performance of TVS in the detection of ureteral DIE, as mentioned in other reports (19, 20).

### Broad Ligament DIE

The diagnostic performance of TVS for lesions involving the broad ligament was not satisfactory in the study. Indeed, to date it has been difficult to find reports on this type of lesion, which can explain the difficulty in diagnosing broad ligament endometriosis to some extent. The main reason may be that the broad ligament has a wide range and suffers from inevitable intestinal disturbance, which makes it difficult to scan fully with TVS. According to our experience, for patients with DIE, if there is an irregular hypoechoic lesion near the uterus, thickened and echo-enhanced surrounding peritoneum, the possibility of broad ligament involvement should be considered.

## Limitations and Future Planning

There are also limitations of our study. First, only patients with severe pelvic endometriosis and surgical evidence of DIE were included, which may have increased the diagnostic rate, and thus we cannot comment on the accuracy of TVS for diagnosing DIE in the general population. Second, there were only a few cases of bladder involvement. Third, the study was carried out only by an experienced doctor. We plan to train low-skilled doctors and discuss the comparison of the accuracy of DIE diagnosis by doctors with different working experience in follow-up research. We also intend to compare the diagnostic performance between MRI and TVS when carrying on the follow-up prospective research. Last but not least, the diagnostic performance of TVS for lesions with broad ligament involvement was not satisfactory, and further study is needed to improve the diagnostic accuracy of TVS for these lesions in the future.

## Conclusions

The results of this study indicate that TVS has great diagnostic value for the diagnosis of pelvic DIE, especially lesions with USL, intestine, and VRS involvement. Understanding the anatomical structure of the involved sites and the characteristics of DIE lesions, the application of the examination ultrasound window and the tenderness guidance method, and the establishment of examination and operation specifications can help improve the rate of ultrasound diagnosis.

## Data Availability Statement

The raw data supporting the conclusions of this article will be made available by the authors, without undue reservation.

## Ethics Statement

The studies involving human participants were reviewed and approved by Ethics committee of Shenzhen People's Hospital. The patients/participants provided their written informed consent to participate in this study.

## Author Contributions

All authors listed have made a substantial, direct and intellectual contribution to the work, and approved it for publication.

## Conflict of Interest

The authors declare that the research was conducted in the absence of any commercial or financial relationships that could be construed as a potential conflict of interest.

## Abbreviations

DIE: deep infiltrating endometriosis
LR+: positive likelihood ratio
LR–: negative likelihood ratio
NPV: negative predictive value
PPV: positive predictive value
SE: sensitivity
SP: specificity
TVS: transvaginal ultrasonography
USL: uterosacral ligament
VRS: vaginal rectal septum.
